# Serum soluble-Fas is a predictor of red blood cell transfusion in critically ill patients

**DOI:** 10.1590/S1679-45082013000400012

**Published:** 2013

**Authors:** Ilana Levy Korkes, Gustavo Schvartsman, Ilson Jorge Lizuka, Beata Marie Quinto, Maria Aparecida Dalboni, Maria Eugênia Canziani, Sergio Antonio Draibe, Virgilio Gonçalves Pereira, Bento Fortunato Cardoso dos Santos, Julio Cesar Martins Monte, Marcelino de Souza Durão, Marcelo Costa Batista, Oscar Fernando Pavão dos Santos, Miguel Angelo de Góes, Miguel Cendoroglo

**Affiliations:** 1 Universidade Federal de São Paulo, São Paulo, SP, Brazil; 2 Hospital Israelita Albert Einstein, São Paulo, SP, Brazil

**Keywords:** Anemia, Inpatients, Erythrocyte transfusion, Critical illness, Hemoglobins, Intensive care units

## Abstract

**Objective:**

To investigate the relation between the need for red blood cell transfusion and serum levels of soluble-Fas, erythropoietin and inflammatory cytokines in critically ill patients with and without acute kidney injury.

**Methods:**

We studied critically ill patients with acute kidney injury (n=30) and without acute kidney injury (n=13), end-stage renal disease patients on hemodialysis (n=25) and healthy subjects (n=21). Serum levels of soluble-Fas, erythropoietin, interleukin 6, interleukin 10, iron status, hemoglobin and hematocrit concentration were analyzed in all groups. The association between these variables in critically ill patients was investigated.

**Results:**

Critically ill patients (acute kidney injury and non-acute kidney injury patients) had higher serum levels of erythropoietin than the other groups. Hemoglobin concentration was lower in the acute kidney injury patients than in other groups. Serum soluble-Fas levels were higher in acute kidney injury and end-stage renal disease patients. Critically ill patients requiring red blood cell transfusions had higher serum levels of soluble-Fas (5,906±2,047 and 1,920±1,060; p<0.001), interleukin 6 (518±537 and 255+502; p=0.02) and interleukin 10 (35.8±30.7 and 18.5±10.9; p=0.02), better iron status and higher mortality rates in the first 28 days in intensive care unit. Serum soluble-Fas levels were independently associated with the number of red blood cell units transfused (p=0.02). Serum soluble-Fas behaved as an independent predictor of the need for red blood cell transfusion in critically ill patients (p=0.01).

**Conclusions:**

Serum soluble-Fas level is an independent predictor of the need for red blood cell transfusion in critically ill patients with or without acute kidney injury. Further studies are warranted to reconfirm this finding.

## RESUMO

**Objetivo:**

Investigar a relação entre a transfusão de hemácias e os níveis séricos de Fas solúvel, eritropoietina e citocinas inflamatórias em pacientes gravemente enfermos, com e sem insuficiência renal aguda.

**Métodos:**

Os seguintes grupos foram estudados: pacientes gravemente enfermos com insuficiência renal aguda (n=30) e sem insuficiência renal aguda (n=13), pacientes portadores de doença renal crônica terminal em hemodiálise (n=25) e indivíduos saudáveis (n=21). Os níveis séricos de Fas solúvel, eritropoietina, interleucina 6, interleucina 10 e ferro, além da concentração de hemoglobina e de hematócrito, foram analisados em todos os grupos. A associação entre tais variáveis foram estudadas nos pacientes gravemente enfermos.

**Resultados:**

Os níveis séricos de eritropoietina mostraram-se mais elevados nos pacientes gravemente enfermos do que nos dos demais grupos. Concentrações mais baixas de hemoglobina foram documentadas nos pacientes com insuficiência renal aguda em relação aos demais. Níveis séricos mais elevados de Fas solúvel foram observados nos pacientes com insuficiência renal aguda e doença renal crônica terminal. Pacientes gravemente enfermos transfundidos apresentaram níveis séricos mais elevados de Fas solúvel (5.906±2.047 e 1.920±1.060; p<0,001), interleucina 6 (518±537 e 255±502; p=0,02), interleucina 10 (35,8±30,7 e 18,5±10,9; p=0,02) e ferro, além de maior mortalidade em 28 dias. Os níveis séricos de Fas solúvel mostraram-se independentemente associados ao número de transfusões (p=0,02). O nível sérico de Fas solúvel foi um preditor independente da necessidade de transfusão de hemácias em pacientes gravemente enfermos (p=0,01).

**Conclusão:**

O nível sérico de Fas solúvel é um preditor independente da necessidade de transfusão de hemácias em pacientes gravemente enfermos, com ou sem insuficiência renal aguda. Mais estudos clínicos e laboratoriais são necessários para confirmar tal resultado.

## INTRODUCTION

Critically ill patients are at high risk for anemia and have high mortality rates^(1,2)^. Red blood cell transfusion is often required to treat anemia in these patients^(1–3)^.

Anemia develops early in the course of critical illness^(2,3)^ and has several consequences in critically ill patients. Therapeutic options and intensive care unit (ICU) hospitalization are costly^(4)^.

Red blood cell transfusions improve oxygen delivery in critically ill patients, but not oxygen consumption^(4,5)^. The anemia of critical illness is a distinct clinical entity with characteristics similar to that of chronic disease anemia^(4–6)^. Acute kidney injury (AKI), inflammation and erythropoietin (Epo) hyporesponsiveness contribute to the progression of anemia in critical illness^(5,6)^.

Epo is an essential growth factor for erythropoiesis and is produced by renal peritubular cells primarily in response to low oxygen tension^(7,8)^.

Fas (CD95) is a transmembrane glycoprotein in the same family as tumor necrosis factor alpha (TNF-α)^(9)^. CD95 activation is a major mechanism of extrinsic apoptosis in several disease processes, including anemia of inflammation^(9,10)^. Soluble Fas (s-Fas), a protein produced by CD95 alternative splicing, prevents binding of CD95-ligand to CD95 in the membrane of different cell types^(11)^, with resulting CD95 inactivation and anti-apoptotic effects, particularly in leukocytes^(9–12)^.

Increased serum s-Fas levels were demonstrated in chronic kidney disease (CKD) and chronic dialysis patients and were associated with inflammatory markers, anemia and Epo hyporesponsiveness^(13)^. More recently, serum s-Fas levels were shown to be related to anemia in AKI patients^(14)^ and may therefore be associated with the need for red blood cell transfusion in critically ill patients.

## OBJECTIVE

We hypothesized that serum s-Fas levels would predict the need for red blood cell transfusion in critically ill patients. To test this hypothesis, serum levels of s-Fas, Epo, inflammatory cytokines, anemia markers, and the need for red blood cell transfusion were determined and compared in critically ill patients with and without AKI, chronic hemodialysis patients and healthy volunteers. Correlations between these variables in critically ill patients with and without AKI were also investigated.

## METHODS

### Subjects and settings

Forty-three critically ill patients with hemoglobin (Hb) concentrations ≤10g/dL and therefore potential candidates for red blood cell transfusion were prospectively studied.

Hb concentration, hematocrit (Ht), iron status and serum levels of s-Fas, Epo and inflammatory cytokines were measured and compared in critically ill patients with AKI (AKI patients; n=30) and without AKI (non-AKI patients; n=13), patients with end-stage renal disease requiring hemodialysis (ESRD patients; n=25) and healthy volunteers (healthy subjects; n=21). Variables were then compared between AKI patients and the three remaining groups to investigate the impact of renal impairment on serum levels of s-Fas, Epo and inflammatory cytokines.

Critically ill patients (n=43) were treated at the ICU of *Hospital Israelita Albert Einstein* in compliance with institutional guidelines. Inclusion criteria were age ≥18 years and at least 24 hours of ICU admission; exclusion criteria were pregnancy, coagulation disorders, folate and vitamin B12 deficiency, major comorbidities (chronic hepatitis B or C and HIV infection), oncologic diseases and primary anemic conditions. Patients receiving erythropoiesis-stimulating agents were also excluded.

Data on the need for red blood cell transfusions within 28 days of follow-up, and up to discharge from ICU or death were collected from all critically ill patients. In these patients, red blood cell transfusions were indicated when Hb concentration dropped below 7.0g/dL to maintain Hb levels between 7.0 and 9.0g/dL. Patients suffering from septic shock, brain injury or acute coronary syndrome were considered at high risk of anemia-related adverse effects and were transfused whenever Hb concentrations dropped below 9.0g/dL (Hb levels, 9.0 to 11.0g/dL).

ESRD patients on hemodialysis were treated at *Hospital do Rim e Hipertensão* of *Universidade Federal de São Paulo* (UNIFESP). Inclusion criteria were age ≥18 years and ongoing hemodialysis for at least 3 months; exclusion criteria included pregnancy, coagulation disorders, hematologic diseases or major comorbidities (malignancy, conjunctive tissue disease, chronic hepatitis B or C and HIV infection). ESRD patients were dialyzed for 3 to 5 hours 3 times weekly. All ESRD patients were under treatment either with subcutaneous recombinant human erythropoietin, intravenous iron infusion or oral vitamin complexes.

Data on AKI and ESRD respective etiologies were collected. Critically ill patients were staged according to the Acute Physiology and Chronic Health Evaluation scale (APACHE II)^(15)^. Renal function assessment was based on serum creatinine levels and urine output. AKI was defined and staged according to Kidney Disease Improving Global Outcomes (KDIGO) criteria^(16,17)^. The 24-hour water balance of all critically ill patients was calculated upon enrollment. All ESRD patients were oligoanuric.

Informed consent was given by all patients or next-of-kin and all healthy volunteers. The study was approved by the Research Ethics Committees of *Hospital Israelita Albert Einstein* and UNIFESP.

### Laboratory workup

Blood samples were collected from all subjects upon enrollment. ESRD patients were sampled before the first hemodialysis session of the week and critically ill patients 5.5±4.3 days after ICU admission. Healthy volunteers were required to fast for a minimum of 8 hours prior to blood sampling. Blood samples were placed in ice and centrifuged within 1 hour of collection. Serum samples were then stored at −80°C until analysis.

Serum levels of s-Fas, interleukin-10 (IL-10) and 6 (IL-6 - BD Biosciences-Pharmigen^®^, San Diego, CA, USA) and Epo (R&D systems^®^, Minneapolis, USA) were measured using enzyme-linked immunoabsorbent assay (ELISA). Serum ferritin levels were measured with the immunofluorimetric assay (IFMA; AxSYM, Abbott^®^, North Chicago, IL, USA).

Serum creatinine, urea and iron levels, transferrin saturation, Hb concentrations and Ht were determined using standard automated laboratory techniques.

### Statistical analysis

Logarithmic conversion was used to compare non-normally distributed variables (serum levels of inflammatory cytokines, s-Fas and Epo, iron status parameters, red blood cell units transfused and diuresis). Continuous variables were expressed as means±SD or percentages. Comparisons between groups were performed using analysis of variance (ANOVA) and the χ^2^ tests. The Tukey test was employed to determine which groups in the sample differed following identification of significant differences among groups (ANOVA). Linear regression (Pearson's coefficient) was used in univariate analysis.

Additional comparisons between both groups of critically ill patients (AKI and non-AKI) and between critically ill patients who required red blood cell transfusion in ICU and those who did not were performed using the Student's *t* test.

Red blood cell transfusions were treated as dependent variables in binary logistic regression. Serum s-Fas level values were divided by 1,000 to obtain equalized decimal values. Hosmer-Lemeshow goodness-of-fit test and Cox & Snell R2 were employed to assess model fit.

The number of red blood cell units transfused was also treated as a dependent variable in multiple linear regression analysis. Independent variables were included in the model whenever p<0.05 in regression analyzes. Hb concentrations are directly related to the indication of red blood cell transfusion in ICU patients and were therefore excluded from the model. Regression analyses were performed in a forward stepwise fashion.

Differences were considered significant when p<0.05 in two-tailed tests. Statistical calculations were performed using software Statistical Package for the Social Sciences (SPSS), version 20.0 for Windows (SPSS Inc. Chicago, IL).

## RESULTS

Critically ill patients were older than patients in other groups. The male gender predominated across all groups. Sepsis, multifactorial etiology and acute tubular necrosis were the most frequent causes of AKI. ESRD was mostly due to *diabetes mellitus* and hypertension. The 24-hour water balance was higher in AKI patients than non-AKI patients. Serum urea and creatinine levels were highest in ESRD patients. Serum creatinine levels were higher in AKI patients than in healthy subjects. Serum urea levels were higher in AKI patients than in healthy subjects and non-AKI patients. Non-AKI patients had higher urine output than AKI patients. Hb concentrations and Ht were lowest in AKI patients. White blood cell counts were higher in AKI patients than ESRD patients and healthy subjects (Table 1).

**Table 1:** Demographic data and comparisons of baseline laboratory values documented in all groups studied

Variable	AKI n=30	non-AKI n=13	ESRD n=25	Healthy n=21	p value
Age (years)	60±19# *	74±17# *	48±13	47±17	<0.001
Gender (% of males)	70	69	68	59	0.79
Renal disease etiology (%)	Sepsis (38) Multifactorial (35) ATN (27)	NA	*Diabetes mellitus* (39) Hypertension (37) Other (16) CGN (8)	NA	–
24-hour water balance (mL)Ψ	+2090±1426	+1517±1074	NA	NA	0.006
Creatinine (mg/dL)Ψ	1.97±0.84#	1.16±0.63	8.57±2.29# Φ *	0.96±0.16	<0.001
Urea (mg/dL)	135±34.3* #	52±27# Φ *	166±23.3#	26.6±6.01	<0.001
Diuresis (mL)Ψ	490±640	1517±1074	NA	NA	<0.001
Hb (g/dL)	8.6±1.3* #	10.2±1.7Φ #	11.1±1.7#	14.5±1.1	<0.001
Ht (%)	26±4* #	33±6Φ #	33±5#	43±3	<0.001
WBC (thou/μL)	11.6±7.4* #	12.0±5.2* #	7.6±2.3	6.4±1.5	0.005
Ferritin (μg/L)Ψ	1390±710* #	576±717Φ	405±217	95.6±67.7	<0.001
Fe (μg/dL)Ψ	73.6±61.8	30.2±28.4Φ * #	79.2±36.1	93.2±19.4	0.004
Transferrin saturation (%)Ψ	71±46* #	34±27Φ	31±12	28±7	<0.001
Epo (mIU/mL)Ψ	97±117* #	43.3±52.8#	16.8±15.6	6.36±3.71	<0.001
s-Fas (pg/mL)Ψ	4709±2562#	1923±1207Φ *	4806±674#	1147±369	<0.001
IL-6 (pg/mL)Ψ	521±583* #	65.3±69.9Φ * #	6.61±10.9	440±6.38	<0.001
IL-10 (pg/mL)Ψ	29.0±25.1* #	22.3±23.1* #	15.8±65.7	39.6±114	<0.001
RBC transfusions (%)^a^	17 (76)	4 (31)	NA	NA	<0.001
RBC units transfused^a^ Ψ	3.7±3.5	0.2±0.6	NA	NA	0.001
Vasoactive therapy, n (%)^a^	25 (83)	4 (31)	NA	NA	0.001
Mechanical ventilation, n (%)^a^	26 (87)	5 (38)	NA	NA	0.001
Sepsis, n (%)	15 (50)	7 (54)	NA	NA	0.82
APACHE II	32±7	19±11	NA	NA	0.35
Mortality, n (%)^a^	17 (57)	1 (8)	NA	NA	0.007

# Different from healthy subjects;

* different from ESRD patients;

Φ different from AKI patients;

Ψ following log transformation for statistical analysis;

a 28-day follow up. AKI: acute kidney injury; ESRD: end-stage renal disease requiring hemodialysis; ATN: acute tubular necrosis; NA: not applicable; CGN: chronic glomerulonephritis; Hb: hemoglobin; Ht: hematocrit; WBC: white blood cells; Fe: serum iron; Epo: erythropoietin; s-Fas: soluble Fas; IL: interleukin; RBC: red blood cell.

Transferrin saturation and serum levels of ferritin and IL-6 were higher in AKI patients. Serum s-Fas levels were higher in AKI and ESRD patients than in non-AKI patients and healthy subjects. Non-AKI patients had the lowest serum levels of iron. Critically ill patients had higher white blood cell counts and serum levels of Epo and IL-10. Mean APACHE II scores did not differ between AKI and non-AKI patients. AKI patients required more red blood cell transfusions, mechanical ventilation and vasoactive therapy, and had higher mortality rates than non-AKI patients (Table 1).

**Figure 1:**
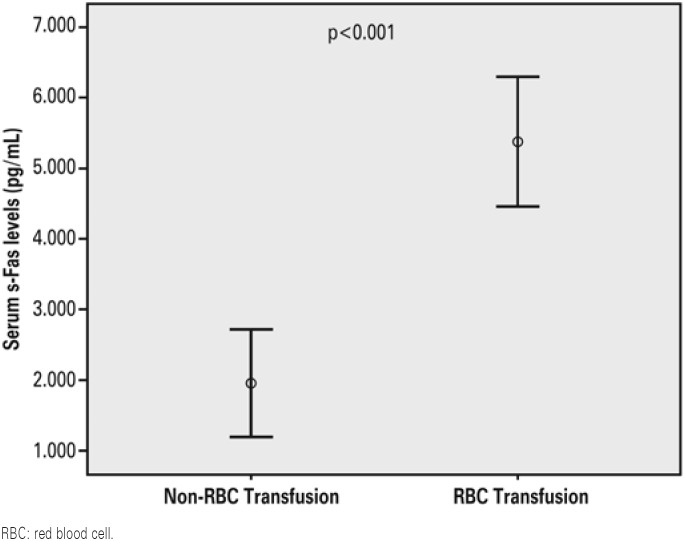
Comparison os serum s-Fas levels between critically ill patient that received and that did not receive red blood cell tranfusion wuthin a 28-day follow-up

Patients requiring red blood cell transfusions had lower Hb concentrations and Ht. Critically ill patients requiring red blood cell transfusions were younger and had higher serum levels of s-Fas (Figure 1), IL-6, iron and ferritin, higher transferrin saturation and higher mortality rates than those that did not require transfusion (Table 2).

**Table 2:** Comparisons of clinical and laboratory data of critically ill patients that required red blood cell transfusion and those that did not (n=43)

Variable	RBC transfusion (n=21)	non-RBC transfusion (n=22)	p value
Age (years)	62±20	66±18	0.55
Gender (% of males)	62	81	0.27
ICU primary admission diagnosis (%)	Sepsis (40) CVD (32) Other (25) Liver disease (3)	Sepsis (39) CVD (28) Other (28) Liver disease (5)	0.36
Diuresis (mL)Ψ	529±654	1,087±1,070	0.49
Creatinine (mg/dL)	1.8±0.7	1.4±0.8	0.29
Hb (g/dL)	7.9±0.9	10.2±1.3	<0.001
Ht (%)	25±3	30±5	<0.001
Transferrin saturation (%)Ψ	76±53	47±29	0.04
Ferritin (μg/L)Ψ	1451±755	919±761	0.03
Fe (μg/dL)Ψ	87±66	38±36	0.007
Epo (mIU/mL)Ψ	103±128	97±99	0.68
RBC units	3.8±3.1	–	NA
s-Fas (pg/mL)Ψ	5,906±2,047	1,920±1,060	<0.001
IL-6 (pg/mL)Ψ	518±537	255±502	0.02
IL-10 (pg/mL)Ψ	35.8±30.7	18.5±10.9	0.02
Mortality (%)	13 (76)	4 (24)	0.02

Ψ following log transformation for statistical analysis. ICU: intensive care unit; CVD: cardiovascular disease; Hb: hemoglobin; Ht: hematocrit; Fe: serum iron; Epo: erythropoietin serum levels; NA: not applicable; s-Fas: soluble Fas; IL: interleukin; RBC: red blood cell.

All AKI patients were in disease stage 3 (KDIGO). ESRD patients had been started on hemodialysis 1.4±0.8 years before enrollment.

**Figure 2:**
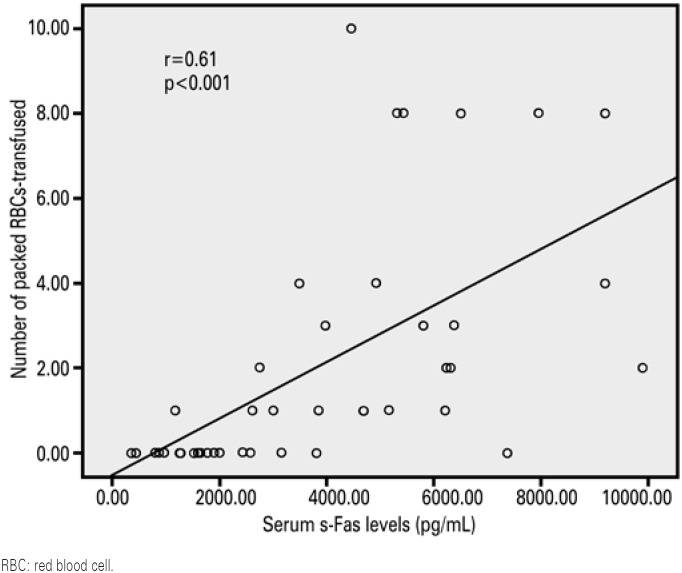
Correlation between serum levels of s-Fas and number of packed red blood cells tranfused

Potential correlations between Hb concentration, serum levels of s-Fas, Epo, inflammatory cytokines and creatinine, the number of red blood cell units transfused and iron status were investigated in critically ill patients. Serum creatinine levels were positively correlated with serum IL-10 levels (r=0.44; p=0.004) and transferrin saturation (r=0.31; p=0.04). Serum Epo levels were positively correlated with serum IL-6 levels (r=0.31; p=0.04) and transferrin saturation (r=0.32; p=0.004). Serum s-Fas levels were positively correlated with serum IL-6 (r=0.63; p<0.001) and ferritin (r=0.46; p=0.002) levels, transferrin saturation (r=0.42; p=0.006), and the number of red blood cell units transfused (Figure 2). The number of red blood cell units transfused was positively correlated with transferrin saturation (r=0.32; p=0.04) and serum levels of IL-6 (r=0.31; p=0.04), ferritin (r=0.42; p=0.006) and iron (r=0.34; p=0.03), and negatively correlated with Hb concentrations (r=-0.36; p=0.02) (Figure 2, Table 2).

Serum s-Fas levels were the only predictors independently associated with red blood cell transfusion in critically ill patients in the first 28 days of follow-up when transferrin saturation and serum levels of ferritin, IL-6 and IL-10 were included in the model (binary logistic regression analysis; Table 3), and the only variable associated with the number of red blood cell units transfused (multiple linear regression; Table 4).

**Table 3:** Results of binary logistic regression analysis using red blood cell transfusion during the first 28 days in intensive care unit as the dependent variable in critically ill patients (n=43)

Variable	WALD test	p value	OR	95%CI Lowest-Highest
s-Fas (pg/mL)	6.582	0.01	1.080	1.065-1.606
IL-10 (pg/mL)	1.527	0.22	1.068	0.962-1.186
IL-6 (pg/mL)	1.061	0.30	0.996	0.988-1.004
Ferritin (μg/mL)	0.908	0.34	0.999	0.996-2.698
Transferrin saturation (%)	0.022	0.88	0.635	0.100-1.793

OR: odds ratio; 95%IC: 95% confidence interval; s-Fas: soluble Fas; IL: interleukin.

**Table 4:** Results of multiple linear regression using the number of red blood cell units transfused as the dependent variable in critically ill patients (n=43)

Variable	Correlation	p value
s-Fas (pg/mL)Ψ	0.62	0.02
Ferritin (μg/L)Ψ	0.20	0.21
IL-6 (pg/L)Ψ	0.18	0.29
Fe (μg/dL)Ψ	0.08	0.74
Transferrin saturation (%)Ψ	−0.07	0.76

Ψ Following log transformation for statistical analysis. s-Fas: soluble Fas; IL: interleukin; Fe: serum iron.

## DISCUSSION

The independent effect of serum s-Fas levels on the indication of red blood cell transfusions in critically ill patients was analyzed in this follow-up study. Previously studied patient groups were evaluated to investigate the associations between Hb concentrations and serum levels of s-Fas, Epo, pro and anti-inflammatory cytokines. The impact of renal function on serum levels of these solutes was also investigated^(13,14)^.

Critically ill patients potentially requiring red blood cell transfusions during the first 28 days in ICU were analyzed. Patients requiring red blood cell transfusions had higher serum levels of s-Fas, IL-6 and IL-10 and better iron status, but also higher mortality rates.

In a binary logistic regression, serum s-Fas levels presented an independent association with red blood cell transfusion after adjusting for the levels of inflammatory cytokines and iron status in critically ill patients. We observed that serum level s-Fas was an independent predictor of red blood cell transfusion and that the addition of each thousand units in serum s-Fas levels increased the chance of needing red blood cell transfusion by eight fold. We also observed that serum s-Fas was an independent predictor of the number of packets of red blood cell transfused in multiple linear regression.

Anemia is a common condition in critically ill ICU patients^(2,18)^ and the risks associated with allogeneic red blood cell transfusions have been well documented^(19,20)^. In these patients, red blood cell transfusions are administered to increase oxygen delivery and tissue oxygenation, as red blood cells are the main transport mechanism for oxygen^(21)^. In a large multicenter cohort study performed in the United States, 44% of critically ill patients received one or more red blood cell units while in ICU. In a European study, a 37% transfusion rate was reported in ICU patients^(2,5)^. In the present study, the higher percentage (49%) of critically ill patients requiring red blood cell transfusions may have reflected the characteristics of the group studied (*e.g.* critically ill patients potentially requiring red blood cell transfusions during the first 28 days in ICU).

In this study, serum IL-6 levels were correlated with serum s-Fas and Epo levels in critically ill patients receiving red blood cell transfusions. Transfused patients in this group had more severe organ dysfunctions and higher serum levels of inflammatory cytokines, and therefore required red blood cell transfusions more often than the other patients studied. The association between serum s-Fas levels and the need for red blood cell transfusion due to anemia in critically ill patients has not been previously reported.

Higher serum s-Fas levels in patients with acute kidney injury and chronic kidney disease requiring renal replacement therapy suggest that serum s-Fas levels may reflect renal function status.

Some caveats must be considered in this study. First and foremost, unclear indications for transfusion of red blood cell may preclude inferences of a causal association between serum s-Fas levels and the need for red blood cell transfusion in critically ill patients. Most critically ill patients in this study suffered from AKI and were on renal replacement therapy, with expected impaired plasma clearance of some solutes. Also, the association between serum s-Fas levels and red blood cell transfusion could not be assessed in all groups due to the small number of subjects involved. Finally, the ability of serum s-Fas levels to predict the actual need for red blood cell transfusion in critically ill patients was based on a short (28 days) follow-up period.

## CONCLUSION

Serum s-Fas levels were associated with the need for red blood cell transfusions in critically ill patients in this study. critically ill patients requiring red blood cell transfusion had higher serum levels of s-Fas and IL-6, better iron status and higher mortality rates. S-Fas may play a role in the pathogenesis of anemia and may be used as a surrogate marker to predict the need for red blood cell transfusion in critically ill patients. Large randomized clinical cohort studies are warranted to further demonstrate the association between serum s-Fas levels and need for red blood cell transfusion in critically ill patients.
